# Ultrasound-based deep learning radiomics nomogram for risk stratification of testicular masses: a two-center study

**DOI:** 10.1007/s00432-023-05549-6

**Published:** 2024-01-19

**Authors:** Fuxiang Fang, Yan Sun, Hualin Huang, Yueting Huang, Xing Luo, Wei Yao, Liyan Wei, Guiwu Xie, Yongxian Wu, Zheng Lu, Jiawen Zhao, Chengyang Li

**Affiliations:** 1 Department of Urology, The First Affiliated Hospital of Guangxi Medical University, 6 Shuangyong Road, Nanning, 530021 China; 2 Department of Emergency, The First Affiliated Hospital of Guangxi Medical University, Nanning, 530021 China; 3 Department of Epidemiology and Health Statistics, School of Public Health of Guangxi Medical University, Nanning, 530021 China; 4 Department of Ultrasound, The First Affiliated Hospital of Guangxi Medical University, Nanning, 530021 China; 5 Department of Urology, Baise People’s Hospital, Baise, 533099 China

**Keywords:** Ultrasound, Testicular lesions, Radiomics, Deep learning

## Abstract

**Objective:**

To develop an ultrasound-driven clinical deep learning radiomics (CDLR) model for stratifying the risk of testicular masses, aiming to guide individualized treatment and minimize unnecessary procedures.

**Methods:**

We retrospectively analyzed 275 patients with confirmed testicular lesions (January 2018 to April 2023) from two hospitals, split into training (158 cases), validation (68 cases), and external test cohorts (49 cases). Radiomics and deep learning (DL) features were extracted from preoperative ultrasound images. Following feature selection, we utilized logistic regression (LR) to establish a deep learning radiomics (DLR) model and subsequently derived its signature. Clinical data underwent univariate and multivariate LR analyses, forming the "clinic signature." By integrating the DLR and clinic signatures using multivariable LR, we formulated the CDLR nomogram for testicular mass risk stratification. The model’s efficacy was gauged using the area under the receiver operating characteristic curve (AUC), while its clinical utility was appraised with decision curve analysis(DCA). Additionally, we compared these models with two radiologists' assessments (5–8 years of practice).

**Results:**

The CDLR nomogram showcased exceptional precision in distinguishing testicular tumors from non-tumorous lesions, registering AUCs of 0.909 (internal validation) and 0.835 (external validation). It also excelled in discerning malignant from benign testicular masses, posting AUCs of 0.851 (internal validation) and 0.834 (external validation). Notably, CDLR surpassed the clinical model, standalone DLR, and the evaluations of the two radiologists.

**Conclusion:**

The CDLR nomogram offers a reliable tool for differentiating risks associated with testicular masses. It augments radiological diagnoses, facilitates personalized treatment approaches, and curtails unwarranted medical procedures.

**Supplementary Information:**

The online version contains supplementary material available at 10.1007/s00432-023-05549-6.

## Introduction

The incidence rate of testicular tumors, which account for approximately 1% of all male tumors and 5% of urinary system tumors, has increased in recent decades, particularly among young and middle-aged men (Park et al. 2018; Znaor et al. 2020; Gurney et al. 2019). The primary symptom is painless testicular enlargement. However, sometimes, they present with symptoms or imaging resembling orchitis, tuberculosis, or other tumor-like conditions, complicating clinical differential diagnosis (Belfield and Findlay-Line 2022; Tandstad et al. 2016). For non-neoplastic testicular lesions, conservative treatment is typically the first approach. However, testicular malignancies often require radical orchiectomy. Studies have shown that unilateral orchiectomy can result in infertility, sexual dysfunction, and reduced sexual function (Henriques et al. 2022; Kerie et al. 2021). Recently, some studies suggest that benign testicular tumors smaller than 2–3 cm in diameter can have a favorable prognosis with partial orchiectomy and adjuvant radiotherapy (Fankhauser et al. 2021; Paffenholz et al. 2018; Gentile et al. 2020; Sm et al. 2023). Thus, preoperative risk assessment of testicular masses is crucial. Accurately differentiating between malignant tumors, benign tumors, and non-neoplastic lesions before treatment ensures the best treatment plan for patients. This strategy prevents over-treatment and unnecessary complete resection, prioritizing the preservation of organ function. Ultrasound is essential in evaluating testicular lesions because of its cost-effectiveness, convenience, high reproducibility, and lack of radiation exposure (Minhas et al. 2021). It offers detailed information about a tumor’s location, size, shape, and blood supply (Lai et al. 2023). However, the varied ultrasound characteristics of testicular masses can challenge diagnosis (Marko et al. 2017).

Radiomics technology, a recent advancement in clinical methods, is proving invaluable for diagnosing, selecting treatments, and assessing the prognosis of patients with tumors (Zhang et al. 2023). It utilizes quantitative analysis techniques to extract extensive lesion information from conventional medical images, conducting in-depth exploration and analysis of medical images to reveal hidden, intricate details within the images (Lafata et al. 2022). Earlier studies have investigated its use in predicting testicular and other urinary system diseases (Santi et al. 2022; Fan et al. 2022; Xue et al. 2023; Baessler et al. 2020). Lately, deep learning (DL) algorithms have gained widespread recognition and adoption in the field of medical image analysis (Beuque et al. 2023; Tong et al. 2022). DL employs neural networks for feature extraction, enabling automated image analysis post-training—a significant advantage over radiomics. Scholars propose merging DL network output with radiomics features, potentially enhancing image-based radiomics' accuracy and reliability, especially with limited training datasets (Zhang et al. 2022). Among the DL algorithms, convolutional neural networks, with their inherent data-driven modeling capabilities, can directly extract task-related features from medical images, thereby significantly enhancing model accuracy and diagnostic efficiency (Yu et al. 2023; Dominique et al. 2022). Yet, there is a current gap in research that merges DL with ultrasound radiomics to predict the risk stratification of testicular masses.

Hence, we introduced two clinical deep learning radiomics (CDLR) nomograms to evaluate their capability in distinguishing between tumors and non-neoplastic lesions, and in differentiating malignant tumors from benign lesions.

## Materials and methods

### Research subjects

**Fig. 1:**
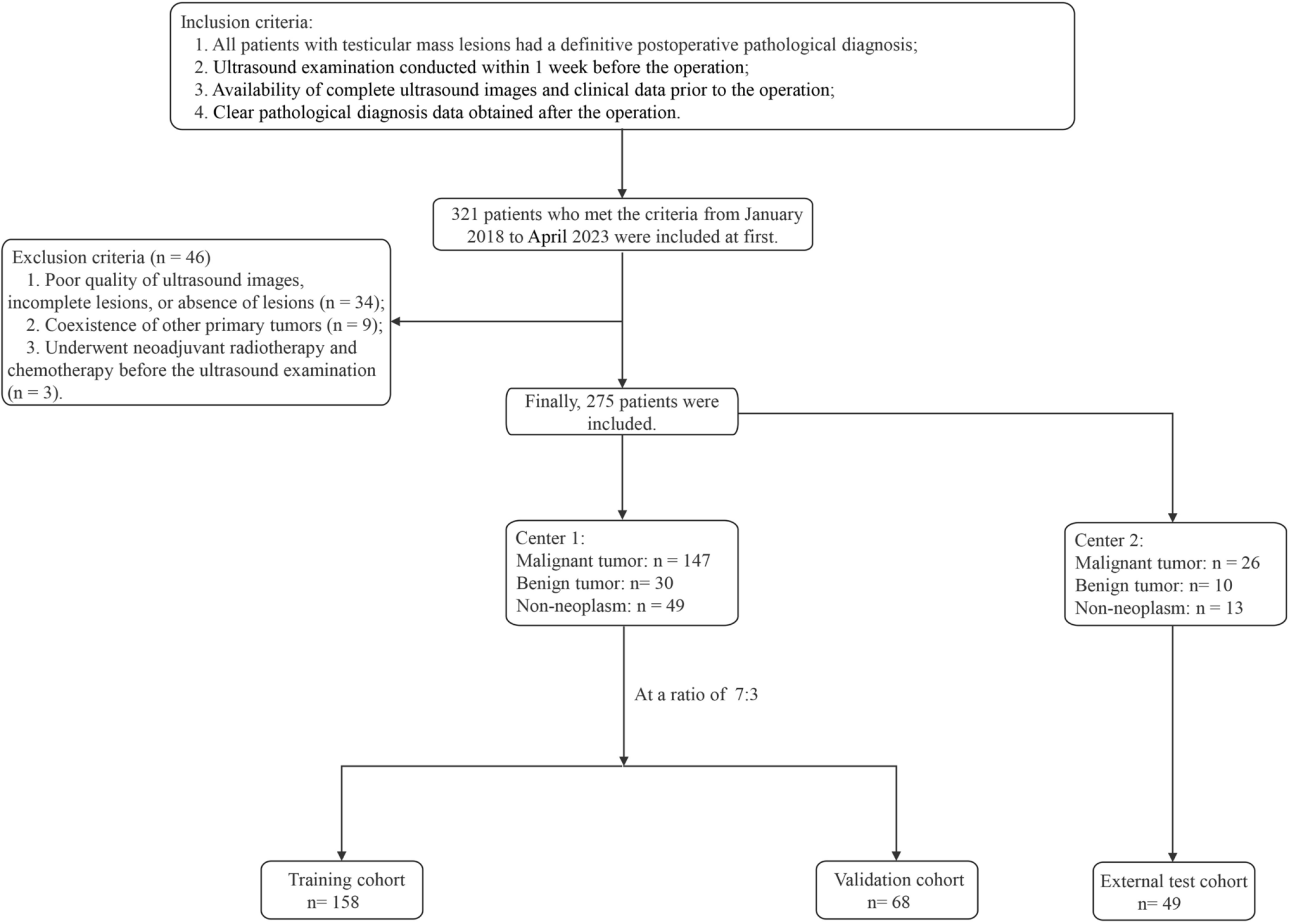
Patient selection process for this study depicted in a flowchart

Having received approval from the Ethics Review Committee and a waiver for patient informed consent, we undertook a retrospective study of 275 patients (275 lesions) diagnosed with testicular space-occupying lesions from January 2018 to April 2023. These patients, representing 275 lesions, were treated at both the First Affiliated Hospital of Guangxi Medical University (Center 1) and Baise People’s Hospital (Center 2). To qualify for the study, patients needed to meet certain inclusion criteria. They must have undergone ultrasound examinations within 1 week before surgery, had full ultrasound images and clinical records pre-surgery, and received definitive postoperative pathological diagnoses. Exclusions involved cases with inferior ultrasound image clarity, no evident lesions, concurrent primary tumors elsewhere, or those who underwent neoadjuvant treatment before their ultrasound. Of the participants, 226 from Center 1 were randomly divided into the training (n = 158 patients) and validation (n = 68 patients) cohorts at a 7:3 ratio. The remaining 49 patients from Center 2 formed an external test cohort. The distribution of lesion pathology types is presented in Supplementary Table 1. An overview of our research process is depicted in Fig. 1.

### Clinical data

The collated clinical information included demographics and health metrics such as age, body mass index (BMI), symptom (scrotal pain), existing medical conditions (e.g., hypertension, diabetes, coronary heart disease), complete blood count, serum alpha-fetoprotein (AFP) levels, serum beta-human chorionic gonadotropin (β-HCG) levels, and more. Radiological evaluations were conducted by experienced radiologists (with 5–8 years under their belts). They meticulously analyzed the ultrasound imagery, gauging lesion blood flow distribution through the Adler grading system. The blood flow was then categorized as either sparse (grades 0–1) or abundant (grades 2–3) based on color Doppler ultrasound readings (Adler et al. 1990; Ma et al. 2015). All clinical data was retrospectively retrieved from the hospital's HIS system.

### Image acquisition

The equipment differed between the two centers. Center 1 utilized the ESAOTE-PLUS color Doppler ultrasound diagnostic equipment from Parkson Medical Company, boasting a high-frequency linear array probe with a 12-MHz frequency. By contrast, Center 2 implemented the Siemens Acuson Sequoia 512 color Doppler ultrasound diagnostic device, outfitted with a 10L4 linear array probe that covered frequencies in the range of 2.9–9.9 MHz. Skilled radiologists, each with more than 5 years of experience, captured the ultrasound images in both institutions. For uniformity, the most expansive cross-sectional lesion view was chosen and saved in the digital imaging and communications in medicine format, accumulating 275 images in total. All images were obtained from the hospital's picture archiving and communication system (PACS) and stored in digital imaging and communications in medicine (DICOM) format.

### Image segmentation and feature extraction

We imported all images into ITK-SNAP software (version 3.8; http://www.itksnap.org). The region of interest (ROI) for each lesion was manually outlined along the edge of the lesion within the software by a radiologist with 5 years of experience. To ensure reliability, we evaluated the reproducibility of the outlined features using both intraclass and interclass correlation coefficients (ICCs). To do this, 30 images were selected at random. A radiologist with 8 years of experience outlined the ROI on these images and, after a week, repeated the process for intra-observer consistency assessment. The both radiologists were blinded to the patients’ clinical information and pathology results.

**Fig. 2:**
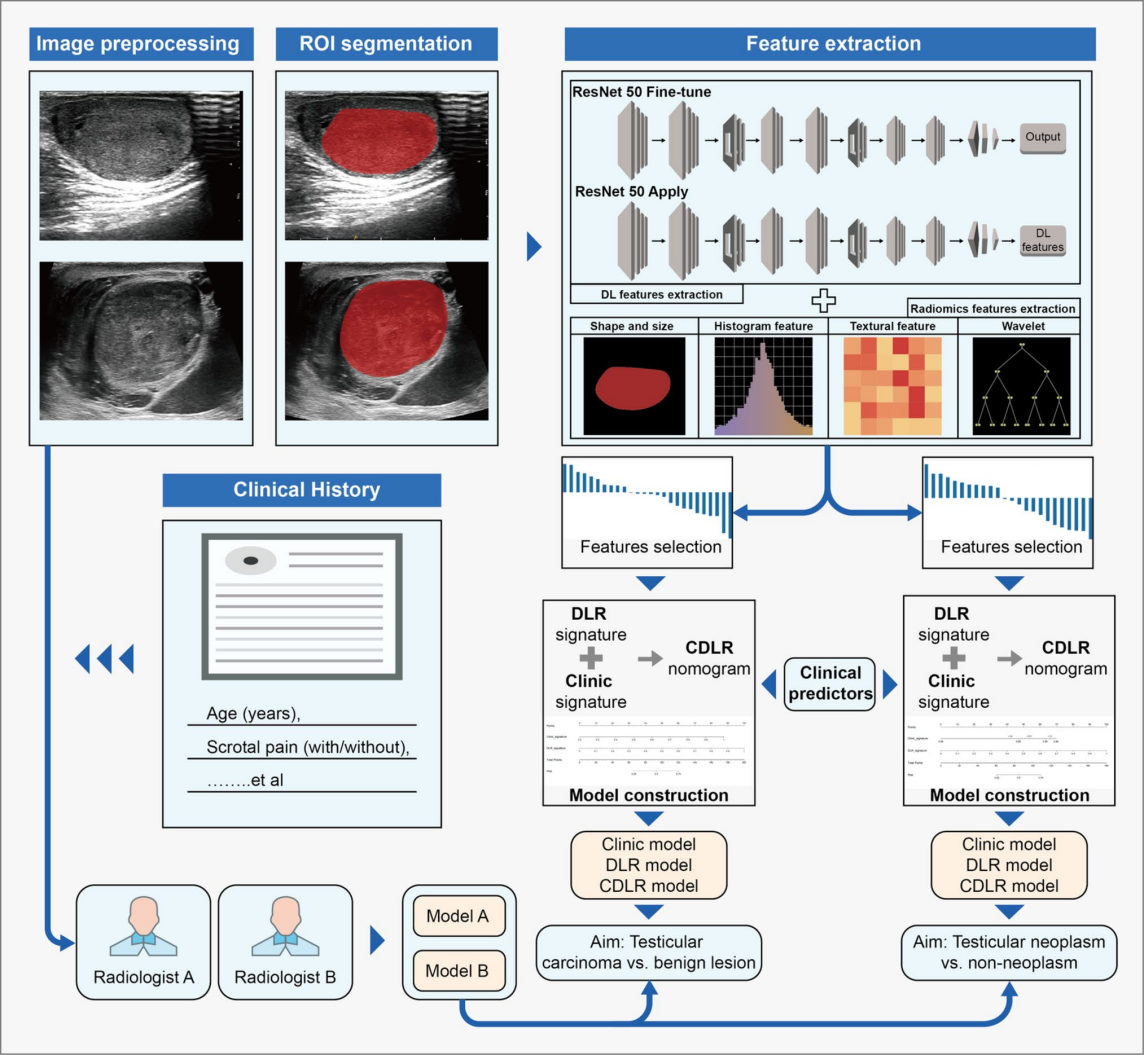
Study workflow of the clinical, DLR, and CDLR models for the risk stratification of testicular masses. *DLR* deep learning radiomics; *CDLR* clinical deep learning radiomics

We extracted radiomics features from these ROIs using the python pyradiomics (https://pyradiomics.readthedocs.io/en/latest/) package. This included (1) fourteen 2D shape-based features, (2) 306 first-order features, (3) texture features, including features from gray level co-occurrence matrix (GLCM) (n = 374), gray-level dependence matrix (GLDM) (n = 238), gray-level run length matrix (GLRLM) (n = 272), gray-level size zone matrix (GLSZM) (n = 272), and neighboring gray tone difference matrix (NGTDM) (n = 85), yielding a total of 1,561 radiomics features (Supplementary material Fig. 1). For the extraction of DL features, we utilized a pre-trained ResNet 50 network on the ImageNet database (https://image-net.org/). The training and validation sets remained consistent before training. We fine-tuned model parameters using a 0.01 initial learning rate, 50 epochs, and a batch size of 32, all processed with a stochastic gradient descent optimizer. The output of the ResNet 50 average pooling layer, with adjusted parameters, helped us obtain 2,048 DL features from the ROI of each patient’s ultrasound image. Figure 2 illustrates our research workflow.

### Feature selection

For the training cohort, we employed a sequential approach to feature screening and dimensionality reduction: First, we retained radiomics features with an ICC exceeding 0.75 and integrated them with the DL features. Then, all selected features were regularized. Second, we applied the minimum redundancy maximum correlation algorithm to further refine feature selection. Finally, using the Least Absolute Shrinkage and Selection Operator (LASSO) regression model along with a tenfold cross-validation process, we identified and retained features with non-zero values. LASSO’s inherent ability for powerful shrinkage and addressing multicollinearity significantly bolstered the accuracy of the model (Liu et al. 2023).

### Establishment of DLR and clinical models

We used LR to construct our models. After the steps of feature screening and dimensionality reduction, we utilized the remaining features to create a DLR model, leading to the generation of a DLR signature. Additionally, single-factor LR analysis was conducted on the clinical characteristics of the training cohort for each variable. If a variable met the significance threshold of p < 0.05, it was chosen for multi-factor LR analysis. This process enabled us to pinpoint critical predictive variables, facilitating the construction of a clinical model. From this, we derived the odds ratio (OR) and their 95% confidence intervals, resulting in a clinical signature.

### Establishment of CDLR

Aiming to fuse clinical and imaging data to develop a precise, objective, and reliable decision-support model, we combined both the clinical and DLR signatures. Using multivariable LR analysis, a combined dimensional CDLR was formulated. For validation, two seasoned radiologists—with 5 and 8 years of ultrasound diagnostic experience—reviewed patient ultrasound images from the validation and test cohorts without knowledge of the pathology. They developed two separate ultrasound feature models, termed Model A and Model B. To gauge the efficacy of these models, receiver operating characteristic (ROC) curves were generated for the training, validation, and test cohorts. From these curves, we determined metrics including AUC, accuracy, sensitivity, specificity, positive predictive value, and negative predictive value. The Delong test was used to discern differences in AUC between models, with a significance level set at p < 0.05. This meticulous evaluation ensures the robustness of our CDLR as a decision-support tool.

### Statistical analysis

For our statistical assessments, we leveraged several software tools, including SPSS software (version 26.0), R software (version 3.6.3; https://www.r-project.org), and Python software (version 3.5.6; http://www.python.org). Descriptive statistics were conveyed as mean ± standard deviation. Differences between cohorts were identified using independent sample t-tests. When data displayed a skewed distribution (Q1, Q3), the Mann–Whitney U test was applied. Ratios for categorical variables were derived from the chi-square or Fisher’s exact test, while skewed count data were subjected to rank sum tests. Both univariate and multivariate LR analyses were performed, with a statistical significance threshold of p < 0.05.

## Results

### Clinical characteristics

Our results can be found in Supplementary Table 2. The results indicated no significant differences among the training, validation, and external test cohorts (p < 0.05). In our study, the training, validation, and test cohorts consisted of 158, 68, and 49 patients, respectively. In Supplementary Table 3, within the training cohort, significant differences were observed in several parameters such as age, lymphocyte count (LYMPH), neutrophil-to-lymphocyte ratio (NLR), platelet-to-lymphocyte ratio (PLR), symptom, serum β-HCG, and AFP when comparing patients with testicular neoplastic lesions to those with non-neoplastic lesions (p < 0.05). Additionally, in Supplementary Table 4, there were distinct differences between benign and malignant testicular lesions in terms of symptom, serum AFP, β-HCG levels, and color Doppler blood flow signals (p < 0.05).

### Construction and validation of DLR

**Fig. 3:**
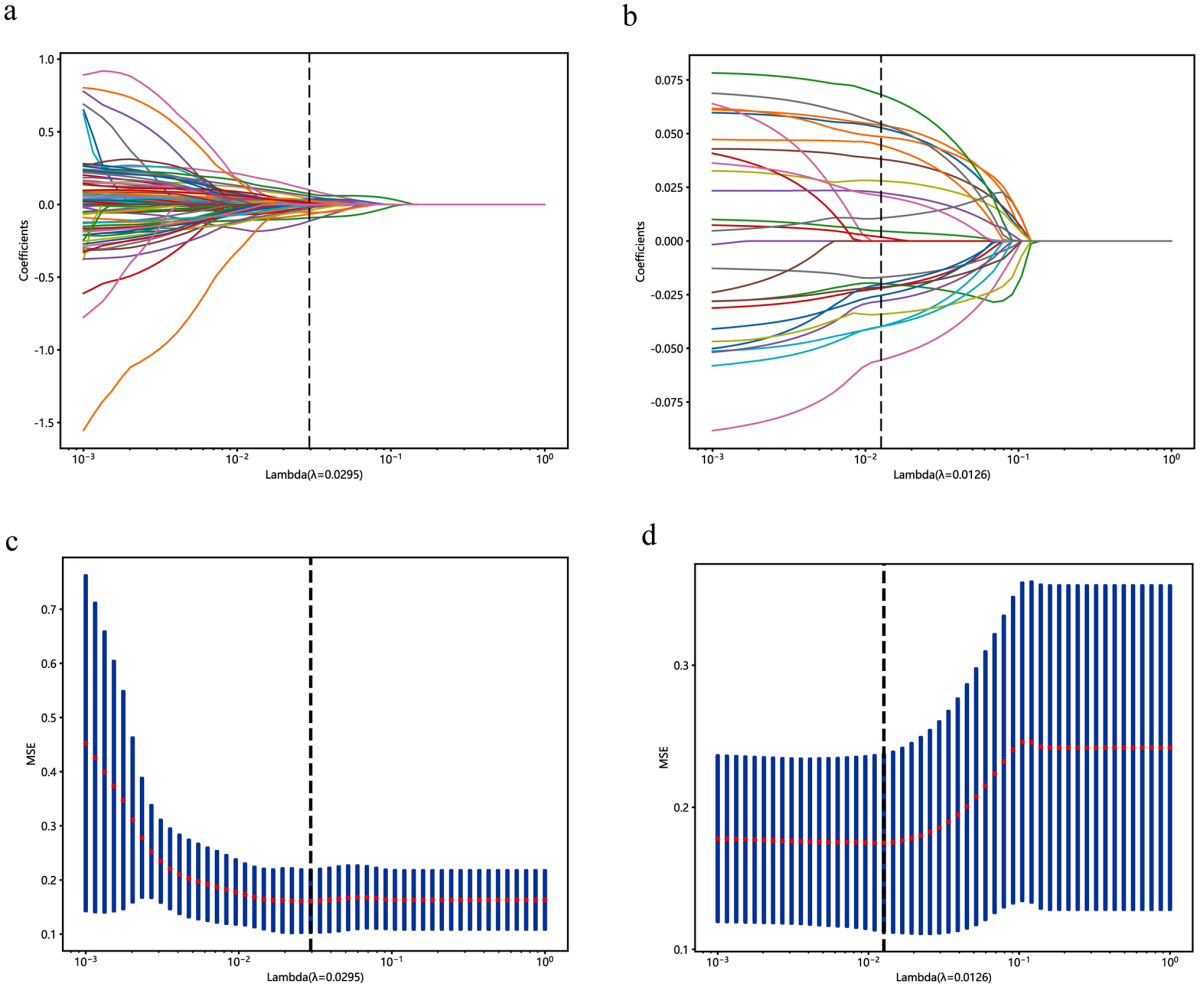
LASSO, paired with ten-fold cross-validation, was employed to screen both radiomics features and DL features for predicting testicular tumors and malignancies. **a**, **b** Show the coefficients of radiomics and DL features obtained from LASSO with ten-fold cross-validation, while **c**, **d** depict the mean squared error (MSE) from the tenfold cross-validation. *LASSO* Least Absolute Shrinkage and Selection Operator; *DL* deep learning; *MSE* mean squared error

**Fig. 4:**
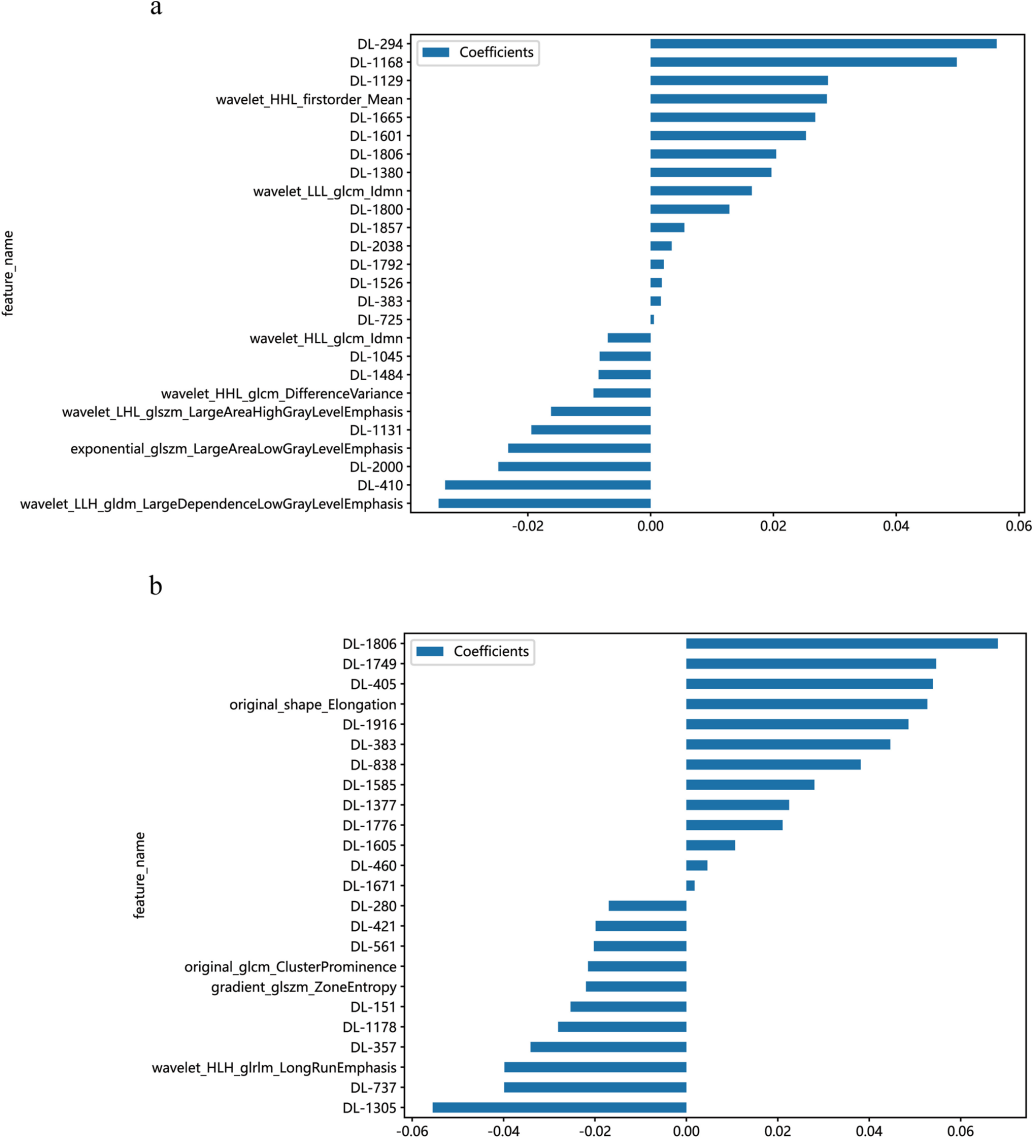
**a**, **b** Coefficients of the filtered radiomics features and DL features. *DL* deep learning

**Fig. 5:**
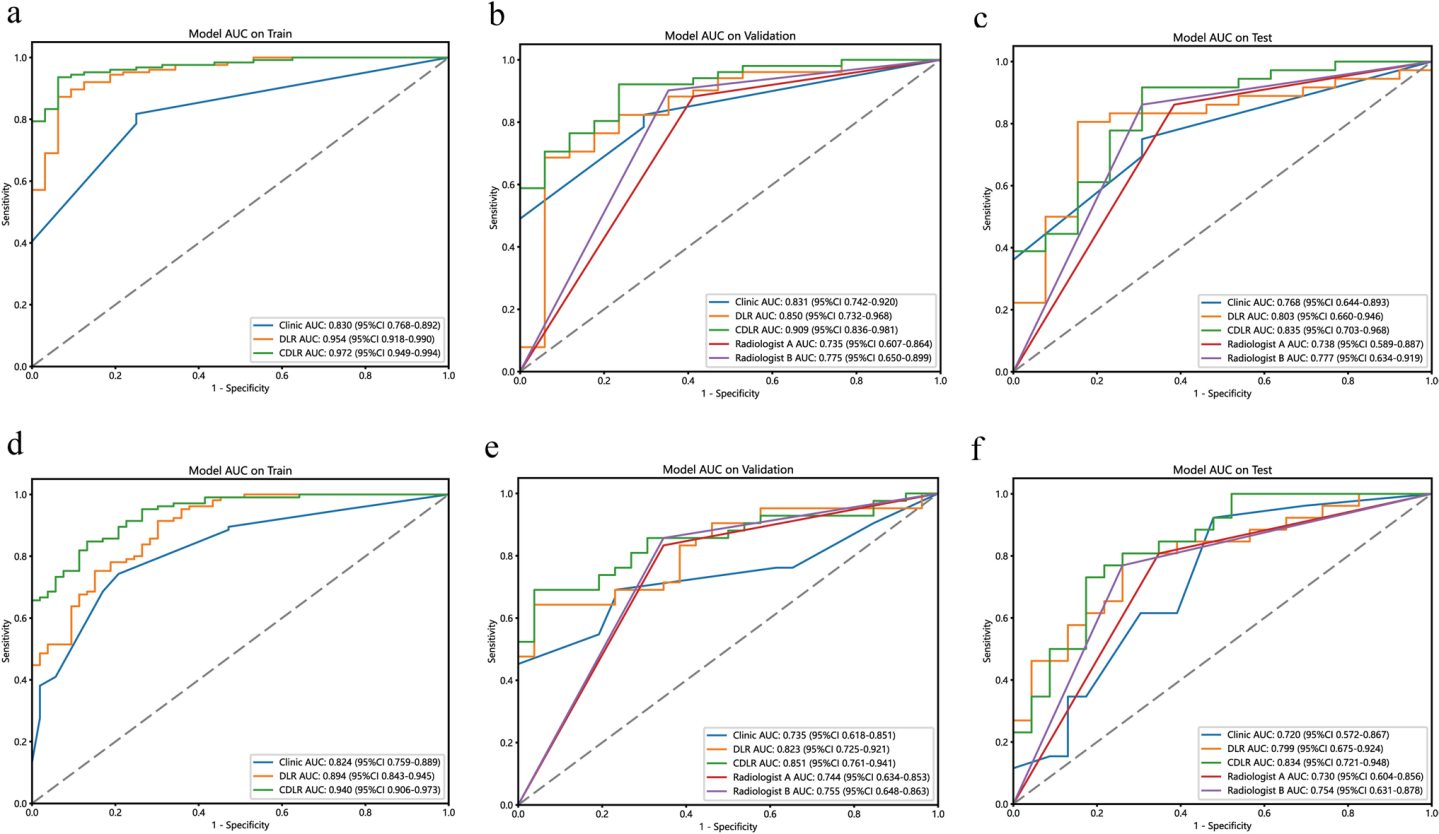
ROC curves comparing different models. **a**–**c** ROC curves comparing the clinical, DLR, and CDLR models for predicting testicular tumors across the training, validation, and test cohorts. **d**–**f** ROC curves comparing the clinical, DLR, and CDLR models for predicting testicular carcinoma in the training, validation, and test cohorts. *ROC* receiver operating characteristic; *DLR* deep learning radiomics; *CDLR* clinical deep learning radiomics

To differentiate testicular tumors from non-tumor lesions, we used 7 radiomics features and 19 DL features to construct the DLR model, from which we derived the DLR signature (Fig. 3a and c, Fig. 4a, Supplementary Table 5). The DLR model’s AUC values for the training, validation, and test cohorts were 0.954, 0.850, and 0.803, respectively (Fig. 5a, b and c). To distinguish between benign and malignant testicular lesions, we employed the same feature selection method, identifying 4 radiomics features and 20 DL features (Fig. 3b and d, Fig. 4b, Supplementary Table 5). This DLR model yielded AUCs of 0.894, 0.823, and 0.799 for the training, validation, and test cohorts, respectively (Fig. 5d, e and f).

### Development and validation of clinical model and CDLR

**Fig. 6:**
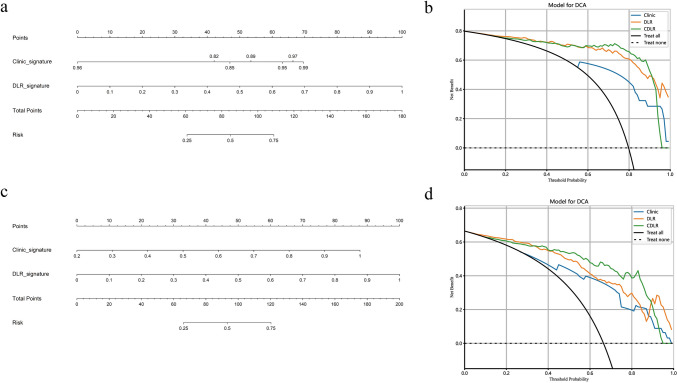
Nomograms and DCA curves; **a** CDLR nomogram for predicting testicular tumors; **b** DCA curve comparison between the clinical, DLR, and CDLR models for predicting testicular tumors. **c** CDLR nomogram for predicting testicular carcinoma; **d** DCA curves comparing clinical, DLR, and CDLR models for predicting testicular carcinoma. *DCA* decision curve analysis; *DLR* deep learning radiomics; *CDLR* clinical deep learning radiomics

**Fig. 7:**
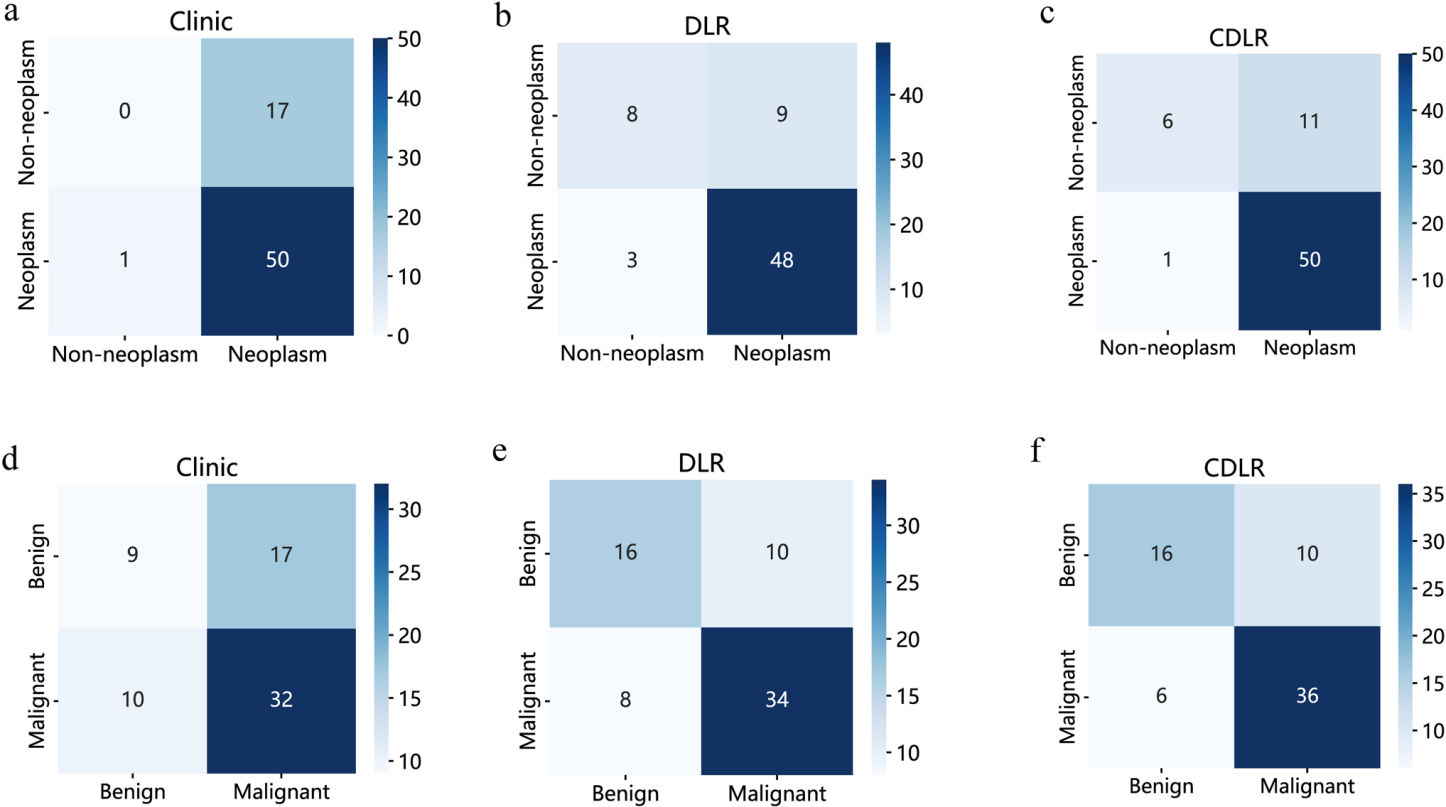
Confusion matrix of different models; **a**–**c** confusion matrix of the clinical, DLR, and CDLR models in the validation cohort for predicting testicular tumors; **d**–**f** confusion matrix of the clinical, DLR, and CDLR models in the validation cohort for predicting testicular carcinoma. *DLR* deep learning radiomics; *CDLR* clinical deep learning radiomics

**Fig. 8:**
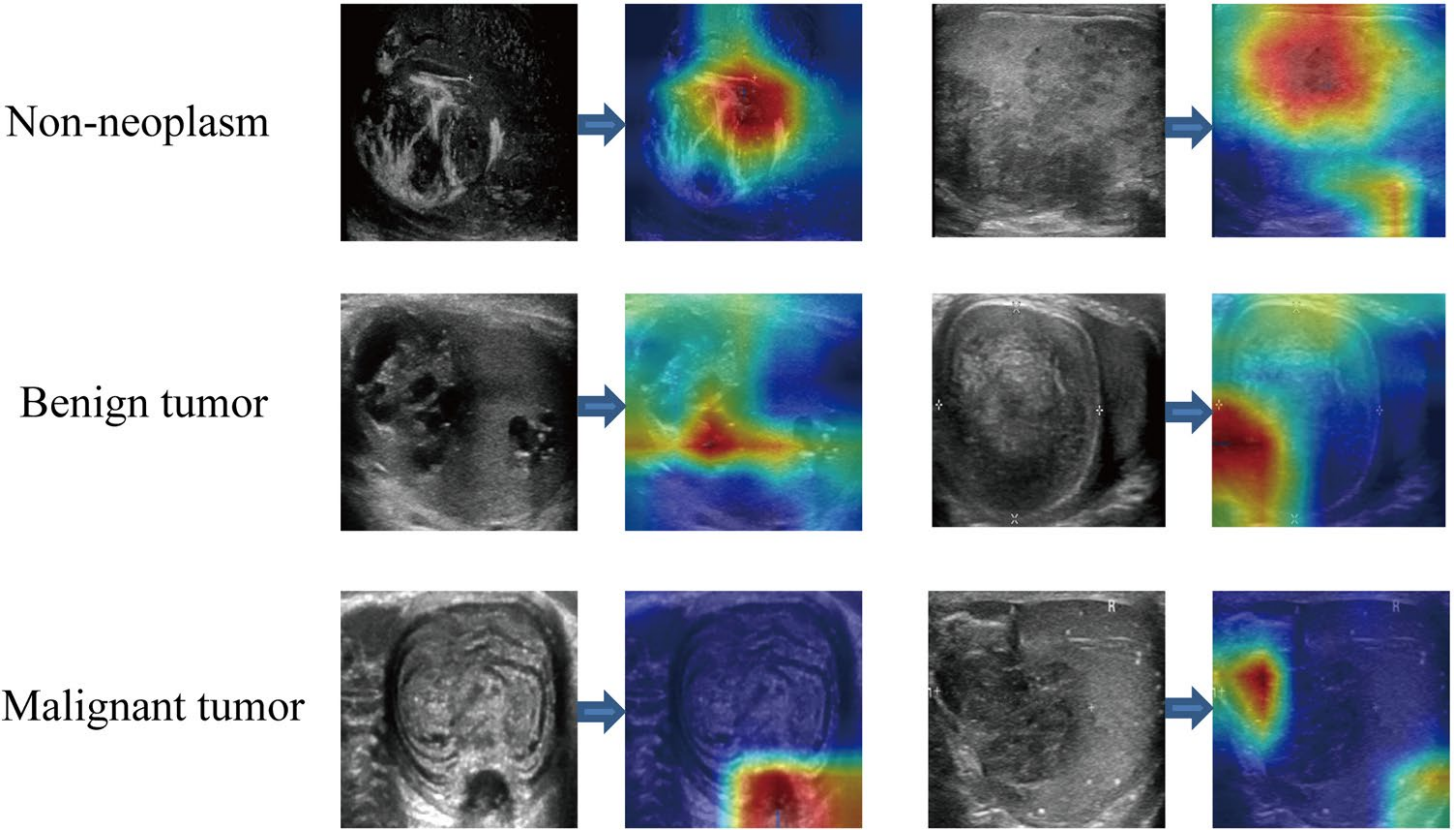
Convolutional neural network activation maps used for identifying testicular non-neoplastic lesions, benign tumors, and malignant tumors. The red regions on these maps highlight areas that correlate with the nature of the mass

Within the training cohort, three independent predictors for testicular neoplastic lesions were identified: the absence of symptom, serum AFP levels ≥ 10 ng/mL, and β-HCG levels ≥ 5 mIU/mL (Supplementary Table 6). From this data, we developed a clinical model, leading to the creation of the clinic signature. By integrating the clinic signature with the DLR signature using multivariable LR, the CDLR showcased enhanced diagnostic prowess (Fig. 5a, b and c, Fig. 6a, Table 1). Specifically, its performance was notably superior to the clinical model (AUC: 0.909 vs. 0.831, p = 0.045), DLR (AUC: 0.909 vs. 0.850, p = 0.211), radiologist A (AUC: 0.909 vs. 0.735, p = 0.041), and radiologist B (AUC: 0.909 vs. 0.775, p = 0.065) in the validation cohort. In the test cohort, CDLR achieved an AUC of 0.835, which exceeded the performances of the clinical model (AUC = 0.768), DLR (AUC = 0.803), and both radiologists (AUC = 0.738 and 0.777, respectively). The absence of symptom, serum AFP ≥ 10 ng/mL, β-HCG ≥ 5 mIU/mL, and color Doppler flow signals (categorized as Adler classification: 2–3) were determined to be independent indicators of testicular malignancy (Supplementary Table 7). In the realm of predicting testicular malignancy (Fig. 5d, e and f, Fig. 6b, Table 2), the CDLR outperformed the clinical model (AUC: 0.851 vs. 0.735, p = 0.014), DLR (AUC: 0.851 vs. 0.823, p = 0.372), radiologist A (AUC: 0.851 vs. 0.744, p = 0.122), and radiologist B (AUC: 0.851 vs. 0.755, p = 0.182) in the validation cohort. Additionally, in the test cohort, CDLR achieved an AUC of 0.834, which outperformed the clinical model (AUC = 0.720), DLR (AUC = 0.799), and radiologist A (AUC = 0.730) and radiologist B (AUC = 0.754). Furthermore, the DCA further indicated that the CDLR delivered more net benefits than the clinical model and DLR in predictions concerning testicular tumors and malignancies (Fig. 6b and d). This superiority in prediction accuracy was further corroborated by the results from the confusion matrix (Fig. 7). Figure 8 displays the activation maps of a convolutional neural network utilized for the identification of testicular non-neoplastic lesions, benign tumors, and malignant tumors.

**Table 1:** Comparison of diagnostic performances of different models for discriminating testicular neoplasm from non-neoplasm in the training, validation, and test cohorts

Model	Cohort	AUC (95% Cl)	Accuracy	Sensitivity	Specificity	PPV	NPV	p value
Clinic	Training	0.830 (0.768, 0.892)	0.804	0.817	0.750	0.928	0.511	< 0.001*
	Validation	0.831 (0.742, 0.920)	0.794	0.824	0.706	0.894	0.571	0.045^#^
	Test	0.768 (0.644, 0.893)	0.735	0.750	0.692	0.871	0.500	0.168^^^
DLR	Training	0.954 (0.918, 0.990)	0.886	0.873	0.938	0.982	0.652	0.044*
	Validation	0.850 (0.732, 0.968)	0.750	0.686	0.941	0.972	0.500	0.211^#^
	Test	0.803 (0.660, 0.946)	0.816	0.806	0.845	0.935	0.806	0.404^^^
CDLR	Training	0.972 (0.950, 0.995)	0.937	0.937	0.938	0.983	0.789	
	Validation	0.909 (0.837, 0.981)	0.882	0.922	0.765	0.922	0.765	
	Test	0.835 (0.703, 0.968)	0.857	0.917	0.692	0.892	0.917	
Radiologist A	Validation	0.735 (0.607, 0.864)	0.809	0.882	0.588	0.865	0.625	0.041^#^
	Test	0.738 (0.589, 0.887)	0.796	0.861	0.615	0.861	0.615	0.309^^^
Radiologist B	Validatin	0.775 (0.650, 0.899)	0.838	0.902	0.647	0.885	0.687	0.065^#^
	Test	0.777 (0.634, 0.919)	0.816	0.861	0.692	0.886	0.643	0.505^^^

*CI* confidence interval; *DLR* deep learning radiomics model; *CDLR* clinical deep learning radiomics nomogram; *PPV* positive predictive value; *NPV* negative predictive value

*p value of AUCs difer between the model and CDLR in the training cohort

^#^p value of AUCs difer between the model and CDLR in the validation cohor

^^^p value of AUCs difer between the model and CDLR in the test cohort

**Table 2:** Comparison of diagnostic performances of different models or discriminating testicular malignant tumor from benign lesions in the training, validation, and test cohorts

Model	Cohort	AUC (95% Cl)	Accuracy	Sensitivity	Specificity	PPV	NPV	p value
Clinic	Training	0.824 (0.759, 0.889)	0.759	0.743	0.792	0.876	0.743	< 0.001*
	Validation	0.735 (0.618, 0.851)	0.721	0.690	0.769	0.829	0.606	0.014^#^
	Test	0.720 (0.572, 0.867)	0.735	0.923	0.522	0.686	0.857	0.046^^^
DLR	Training	0.894 (0.843, 0.945)	0.842	0.914	0.698	0.857	0.804	0.044*
	Validation	0.823 (0.726, 0.921)	0.765	0.643	0.962	0.964	0.625	0.372^#^
	Test	0.799 (0.674, 0.914)	0.653	0.846	0.435	0.629	0.714	0.365^^^
CDLR	Training	0.940 (0.906, 0.974)	0.854	0.848	0.868	0.927	0.742	
	Validation	0.851 (0.761, 0.941)	0.794	0.690	0.962	0.967	0.658	
	Test	0.834 (0.721, 0.948)	0.755	0.808	0.696	0.750	0.762	
Radiologist A	Validation	0.744 (0.634, 0.853)	0.765	0.833	0.654	0.795	0.708	0.122^#^
	Test	0.730 (0.604, 0.856)	0.735	0.808	0.652	0.724	0.750	0.240^^^
Radiologist B	Validatin	0.755 (0.648, 0.863)	0.779	0.857	0.654	0.800	0.739	0.182^#^
	Test	0.754 (0.631, 0.878)	0.755	0.769	0.739	0.769	0.739	0.373^^^

*CI* confidence interval; *DLR* deep learning radiomics model; *CDLR* clinical deep learning radiomics nomogram; *PPV* positive predictive value; *NPV* negative predictive value

*p value of AUCs difer between the model and CDLR in the training cohort

^#^p value of AUCs difer between the model and CDLR in the validation cohor

^^^p value of AUCs difer between the model and CDLR in the test cohort

## Discussion

Our study indicates that the CDLR surpasses the clinical model, DLR, and radiologists with 5–8 years of experience in diagnosing testicular tumors and malignancies. CDLR can be a pivotal tool to support radiologists in imaging diagnosis and help clinicians in making tailored decisions, ultimately cutting down on unnecessary medical procedures.

Correctly diagnosing testicular masses is vital, as treatments range from conservative measures to radical surgery. Overlooking a testicular malignancy diagnosis can cause treatment delays and poorer outcomes. For patients with benign testicular tumors, partial orchiectomy can conserve testicular function (Fankhauser et al. 2021; Paffenholz et al. 2018; Gentile et al. 2020; Sm et al. 2023). Conversely, unneeded surgical resections for those with non-neoplastic testicular lesions can adversely affect androgen levels, sexual function, fertility, among others (Henriques et al. 2022; Kerie et al. 2021). Hence, it’s paramount to study and ascertain the nature of testicular masses to minimize unnecessary surgeries and reduce missed diagnoses of malignancies. To our understanding, we are the first research team to devise and authenticate CDLR nomograms to predict testicular mass risk stratification, targeting the identification of neoplastic lesions and malignancies.

Radiomics is a burgeoning non-invasive diagnostic method in medical imaging, which focuses on extracting a plethora of quantitative traits from comprehensive medical image data and leveraging this for diagnosis and forecasting. This approach is renowned for its objectivity, non-invasiveness, and data-mining capabilities, marking its potential in tumor diagnosis and treatment (Lambin et al. 2012; Guiot et al. 2022). DL is a formidable method in image analysis, facilitating the derivation of profound insights from image datasets. In our research, we employed deep transfer learning to draw DL attributes and merged them with radiomics traits to determine the nature of the masses. When predicting neoplastic lesions and malignant tumors, DL features stood out in terms of volume and significance among the chosen attributes. This observation underscores that DL technology can adeptly pinpoint key quantitative data mirroring the nature of the masses, thus becoming an indispensable tool for precise diagnoses.

Prior research has identified a link between pain and non-neoplastic lesions, with angiogenesis detected via color Doppler ultrasound emerging as a vital independent risk factor for malignancy (Liu et al. 2023). Tumor markers like AFP and β-HCG are instrumental in pinpointing testicular tumors (Esen et al. 2018). Our results concur with these findings; we recognized asymptomatic scrotal conditions and elevated serum AFP or β-HCG levels as standalone predictors of testicular tumors. Moreover, we identified asymptomatic scrotal conditions, increased serum AFP or β-HCG levels, and distinct blood flow signals via color Doppler ultrasound as independent predictors of testicular malignancy. Nevertheless, the accuracy of conventional ultrasound diagnosis for testicular tumors needs enhancement, currently hovering around 76.9% (Lung et al. 2020; Andipa et al. 2004). While contrast-enhanced ultrasound (CEUS) is a newer imaging modality, standard ultrasonography remains the go-to for diagnosing testicular masses (Schröder et al. 2016). The constraints of CEUS—such as the need for specialized expertise, higher costs, limited access, and potential contraindications linked to ultrasound contrast agents—have curbed its broad clinical adoption (Liu et al. 2017). In our research, the CDLR attained a commendable accuracy of 88.2%. Past studies emphasized the difficulty in differentiating benign from malignant testicular masses using only conventional ultrasound (Andiap et al. 2004). Fan et al. leveraged magnetic resonance imaging (MRI) volumetric apparent diffusion coefficient histogram analysis, attaining an AUC of 0.822 (Fan et al. 2020). They then integrated MRI imaging with machine learning, producing a prediction model for testicular masses with an AUC of 0.868 (Fan et al. 2022). The enhanced performance in these studies might stem from the extraction of richer features in the radiomics model. Yet, MRI comes with challenges: it’s less sensitive to calcifications, has patient contraindications, is costlier, and has extended examination durations. Our CDLR, showcasing an AUC of 0.851 and an accuracy rate of 79.4%, underlines its significant advantages and potential in this arena.

In this study, the standalone DLR showcased superior performance compared to the clinical model, highlighting the importance of using deep image information from radiomics and DL to discern the features of testicular masses. When combined with clinical data, the CDLR displayed better predictive capabilities, surpassing even radiologists with 5–8 years of experience. This breakthrough can assist radiologists in precisely identifying testicular tumors and malignancies.

However, this study has some limitations. Firstly, as a retrospective study, selection bias and errors are unavoidable. For instance, if ultrasound examinations are conducted by different doctors, subjective errors might arise when selecting the maximum diameter section of the tumor. Secondly, while our study combines clinical characteristics, imaging, and modeling of radiomics and DL features, it doesn’t include other imaging techniques such as contrast-enhanced ultrasound and elastography for comparison or multi-modal fusion. Lastly, defining the ROI boundary might introduce researcher subjectivity. We anticipate using DL technology for automatic identification and delineation of ROI in the future, and we plan on conducting prospective, multicenter studies to further validate our proposed model.

## Conclusion

The clinical-deep learning ultrasound radiomics nomogram introduced in this study produced encouraging results in predicting testicular tumors and malignancies. It even outperformed radiologists with between 3 and 8 years of professional experience. This is crucial for early patient diagnosis, treatment planning, and surgical method decision-making. It can help prevent unnecessary testicle removal or damage to testicular function from excessive medical intervention, providing solid backing for achieving precise tumor treatment goals.

## Supplementary Information

Below is the link to the electronic supplementary material.

Supplementary file1 (DOCX 162 KB)

## Data Availability

The original contributions presented in the study are included in the article/supplementary material, further inquiries can be directed to the corresponding authors.

## Abbreviations

AUC: Area under the receiver operating characteristic curve
AFP: Alpha-fetoprotein
BMI: Body mass index
β-HCG: Beta-human chorionic gonadotropin
CDLR: Clinical deep learning radiomics
CEUS: Contrast-enhanced ultrasound
CI: Confidence interval
DLR: Deep learning radiomics
DCA: Decision curve analysis
DICOM: Digital imaging and communications in medicine
GLCM: Gray level co-occurrence matrix
GLDM: Gray-level dependence matrix
GLRLM: Gray-level run length matrix
GLSZM: Gray-level size zone matrix
ICC: Interclass correlation coefficient
LR: Logistic regression
LASSO: Least Absolute Shrinkage and Selection Operator
LYMPH: Lymphocyte count
MRI: Magnetic resonance imaging
NGTDM: Neighboring gray tone difference matrix
NLR: Neutrophil-to-lymphocyte ratio
NPV: Negative predictive value
OR: Odds ratio
PAS: Picture archiving and communication system
PLR: Platelet-to-lymphocyte ratio
PPV: Positive predictive value
ROI: Region of interest
ROC: Receiver operating characteristic
