# Supplementary material for: Ultrasound-based deep learning radiomics nomogram for risk stratification of testicular masses: a two-center study
Source: J Cancer Res Clin Oncol. 2024 Jan 19;150(1):18. doi: 10.1007/s00432-023-05549-6 (PMC10798931; doi:10.1007/s00432-023-05549-6)
Supplement: Supplementary file 1 — Supplementary file1 (DOCX 162 KB) [file 432_2023_5549_MOESM1_ESM.docx]

**Supplementary Table 1** Histopathological analysis of testicular lesions.

| Pathological type | Patiens (n) | % |
| --- | --- | --- |
| Malignant tumor | 173 | 62.9% |
| Seminoma | 83 | 30.2% |
| Yolk sac tumor | 30 | 10.9% |
| Mixed germinoma | 19 | 6.9% |
| Embryonal carcinoma | 12 | 4.4% |
| Malignant teratoma | 11 | 4.0% |
| Primary lymphoma | 10 | 3.6% |
| Leiomyosarcoma | 6 | 2.1% |
| Squamous cell carcinoma | 1 | 0.4% |
| Adenocarcinoma | 1 | 0.4% |
| Testicular benign tumor | 40 | 14.6% |
| Benign teratoma | 33 | 12.0% |
| Mesenchymal tumor | 5 | 1.8% |
| Leiomyoma | 2 | 0.8% |
| Non-neoplastic lesions | 62 | 22.5% |
| Chronic granulomatous inflammation | 34 | 12.3% |
| Chronic suppurative inflammation | 16 | 5.8% |
| Acute orchitis | 8 | 2.9% |
| Cyst | 4 | 1.5% |

**Supplementary Table 2** Clinical characteristics in the training, validation and test cohorts.

| Variable | | Training cohort  (n=158) | Validation cohort  (n=68) | External test cohort (n=49) | *P*-value |
| --- | --- | --- | --- | --- | --- |
| Age (years)  BMI (kg/m2)  WBC (10^9^/L) | | 30.23±19.73 | 30.28±21.45 | 30.57±20.90 | 0.995 |
|  |  | 21.43±5.27 | 20.59±3.96 | 21.36±4.68 | 0.481 |
|  |  | 8.60±3.45 | 8.34±4.19 | 7.65±1.92 | 0.237 |
| RBC (10^9^/L) |  | 4.94±0.73 | 4.99±0.87 | 4.96±0.84 | 0.627 |
| HGB (g/L) |  | 130.13±25.53 | 130.05±17.10 | 129.53±25.61 | 0.121 |
| PLT (10^9^/L) |  | 290.50±93.61 | 302.71±103.52 | 265.06±127.46 | 0.144 |
| NEUT (10^9^/L) |  | 5.12±3.09 | 4.66±3.80 | 4.07±1.69 | 0.104 |
| LYMPH (10^9^/L) |  | 2.35±1.52 | 2.67±1.78 | 2.63±1.44 | 0.714 |
| MONO (10^9^/L) |  | 0.71±0.81 | 0.66±0.40 | 0.86±0.3 | 0.216 |
| NLR |  | 3.05±3.50 | 2.69±3.65 | 2.34±2.33 | 0.398 |
| MLR |  | 0.34±0.25 | 0.34±0.39 | 0.37±0.27 | 0.288 |
| PLR |  | 147.06±84.09 | 152.14±93.36 | 142.35±84.20 | 0.198 |
| Symptom | With | 68 (43.0%) | 26 (38.2%) | 24 (49.0%) | 0.511 |
|  | Without | 90 (57.0%) | 42 (61.8%) | 25 (51.0%) |  |
| Basic illness | with | 14 (8.9%) | 6 (8.8%) | 5 (10.2%) | 0.956 |
|  | without | 144 (91.1%) | 62 (91.2%) | 44 (89.8%) |  |
| AFP (ng/mL) | <10 | 110 (69.6%) | 48 (70.6%) | 38 (77.6%) | 0.557 |
|  | ≥10 | 48 (30.4%) | 20 (29.4%) | 11 (22.4%) |  |
| β-HCG (mIU/mL) | <5 | 133 (84.2%) | 52 (76.5%) | 41 (83.7%) | 0.364 |
|  | ≥5 | 25 (15.8%) | 16 (23.5%) | 8 (16.3%) |  |
| Adler classification | 0-1 | 57 (36.1%) | 28 (41.1%) | 19 (39.8%) | 0.760 |
|  | 2-3 | 101 (63.9%) | 40 (58.8%) | 30 (61.2%) |  |

*, *P* < 0.05; BMI, body mass index; WBC, white blood cell count; RBC, red blood cell count; HGB, hemoglobin; PLT, platelet count; NEUT, neutrophil count; LYMPH, lymphocyte count; MONO, monocyte count; NLR, neutrophil-to-lymphocyte ratio; MLR, monocyte-to-lymphocyte ratio; PLR, platelet-to-lymphocyte ratio.

| **Supplementary Table 3** Baseline analysis conducted in the training, validation, and test cohorts based on clinical features. | | | | | | | | | |
| --- | --- | --- | --- | --- | --- | --- | --- | --- | --- |
| Variable | Training cohort (n=158) | | | Validation cohort (n=68) | | | External test cohort (n=49) | | |
|  | Non-neoplasm (n=32) | Neoplasm (n=126) | *P*-value | Non-neoplasm (n=17) | Neoplasm (n=51) | *P*-value | Non-neoplasm (n=13) | Neoplasm (n=36) | *P*-value |
| Age (years) | 42.66±19.52 | 27.08±18.57 | <0.001* | 43.76±25.82 | 25.78±17.92 | 0.007* | 33.15±22.65 | 29.64±20.49 | 0.768 |
| BMI (kg/m^2^) | 22.26±3.27 | 21.22±5.66 | 0.102 | 20.52±2.92 | 20.61±4.28 | 0.972 | 20.56±3.60 | 21.66±5.02 | 0.415 |
| WBC (10^9^/L) | 8.73±4.04 | 8.57±3.31 | 0.626 | 9.08±7.00 | 8.09±2.77 | 0.488 | 7.89±1.92 | 7.56±1.94 | 0.469 |
| RBC (10^9^/L) | 4.83±0.62 | 4.96±0.76 | 0.310 | 4.69±0.57 | 5.09±0.93 | 0.145 | 5.04±0.47 | 15.05±33.82 | 0.518 |
| HGB (g/L) | 127.93±20.52 | 130.68±26.70 | 0.381 | 132.82±14.81 | 129.12±17.83 | 0.630 | 140.19±14.93 | 152.90±52.27 | 0.511 |
| PLT (10^9^/L) | 271.84±95.70 | 295.24±92.86 | 0.279 | 266.82±99.04 | 314.68±103.14 | 0.142 | 277.58±121.32 | 260.54±130.97 | 0.964 |
| NEUT (10^9^/L) | 6.03±4.04 | 4.89±2.77 | 0.263 | 6.48±6.45 | 4.05±2.14 | 0.092 | 4.54±1.83 | 3.90±1.63 | 0.428 |
| LYMPH (10^9^/L) | 1.81±0.75 | 7.50±5.40 | 0.013* | 1.65±0.75 | 3.01±1.89 | 0.002* | 2.47±1.40 | 2.69±1.48 | 0.651 |
| MONO (10^9^/L) | 0.56±0.23 | 0.75±0.89 | 0.060 | 0.78±0.65 | 0.62±0.26 | 0.932 | 0.67±0.26 | 4.19±11.93 | 0.434 |
| NLR | 4.28±4.97 | 2.74±2.97 | 0.003* | 4.96±6.47 | 1.93±1.45 | 0.001* | 2.69±2.73 | 2.21±2.20 | 0.563 |
| MLR | 0.36±0.21 | 0.33±0.26 | 0.122 | 0.58±0.68 | 0.26±0.17 | 0.003* | 0.39±0.36 | 0.37±0.34 | 0.892 |
| PLR | 169.97±85.56 | 141.24±83.05 | 0.008* | 194.52±132.67 | 138.01±72.34 | 0.169 | 135.64±70.81 | 105.60±60.64 | 0.135 |
| Symptom | | | | | | |  |  |  |
| With | 24 (75.0%) | 44 (34.9%) | <0.001* | 12 (70.6%) | 14 (27.5%) | 0.004* | 7 (53.9%) | 17 (47.2%) | 0.932 |
| Without | 8 (25.0%) | 82 (65.1%) |  | 5 (29.4%) | 37 (72.5%) |  | 6 (46.1%) | 19 (52.8%) |  |
| Basic illness | | | | | | |  |  |  |
| With | 5 (15.6%) | 9 (7.1%) | 0.246 | 4 (23.5%) | 2 (3.9%) | 0.048* | 3 (23.1%) | 2 (5.6%) | 0.210 |
| Without | 27 (84.4%) | 117 (92.9%) |  | 13 (76.5%) | 49 (96.1%) |  | 10 (76.9%) | 34 (94.4%) |  |
| AFP (ng/mL) | | | | | | |  |  |  |
| <10 | 32 (100.0%) | 78 (61.9%) | <0.001* | 17 (100.0%) | 31 (60.8%) | 0.006* | 13 (100.0%) | 26 (72.2%) | 0.008* |
| ≥10 | 0 (0%) | 48 (38.1%) |  | 0 (0%) | 20 (39.2%) |  | 0 (0%) | 10 (27.8%) |  |
| β-HCG (mIU/mL) | | | | | | |  |  |  |
| <5 | 32 (100.0%) | 101 (80.2%) | 0.013* | 17 (100.0%) | 35 (68.6%) | 0.021* | 13 (100.0%) | 29 (80.6%) | 0.019* |
| ≥5 | 0 (0%) | 25 (19.8%) |  | 0(0%) | 16 (31.4%) |  | 0 (0%) | 7 (19.4%) |  |
| Adler classification | | | | | | |  |  |  |
| 0-1 | 15 (46.9%) | 42 (33.3%) | 0.223 | 5 (29.4%) | 15 (29.4%) | 1.000 | 6 (46.2%) | 13 (36.1%) | 0.760 |
| 2-3 | 17 (53.1%) | 84 (66.7%) |  | 12 (70.6%) | 36 (70.6%) |  | 7 (53.9%) | 23 (63.9%) |  |
| *, *P* < 0.05; BMI, body mass index; WBC, white blood cell count; RBC, red blood cell count; HGB, hemoglobin; PLT, platelet count; NEUT, neutrophil count; LYMPH, lymphocyte count; MONO, monocyte count; NLR, neutrophil-to-lymphocyte ratio; MLR, monocyte-to-lymphocyte ratio; PLR, platelet-to-lymphocyte ratio. | | | | | | | | | |

| **Supplementary Table 4** Baseline analysis conducted in the training, validation, and test cohorts based on clinical features. | | | | | | | | | |
| --- | --- | --- | --- | --- | --- | --- | --- | --- | --- |
| Variable | Training cohort (n=158) | | | Validation cohort (n=68) | | | External test cohort (n=49) | | |
|  | Benign  (n=53) | Malignant (n=105) | *P*-value | Benign  (n=26) | Malignant (n=42) | *P*-value | Benign  (n=23) | Malignant (n=26) | *P*-value |
| Age (years) | 30.62±22.33 | 30.04±18.39 | 0.861 | 34.85±26.65 | 27.45±17.25 | 0.169 | 30.57±21.88 | 30.58±20.44 | 0.998 |
| BMI (kg/m^2^) | 21.00±4.29 | 21.64±5.71 | 0.467 | 20.38±3.54 | 20.72±4.24 | 0.733 | 20.59±3.32 | 22.05±5.59 | 0.281 |
| WBC (10^9^/L) | 8.36±3.47 | 8.73±3.45 | 0.516 | 8.88±5.74 | 8.88±5.74 | 0.412 | 7.89±2.02 | 7.43±1.85 | 0.402 |
| RBC (10^9^/L) | 4.92±0.60 | 4.95±0.79 | 0.805 | 4.85±0.66 | 5.08±0.98 | 0.297 | 9.89±23.29 | 14.62±33.93 | 0.577 |
| HGB (g/L) | 128.46±20.09 | 130.97±27.93 | 0.562 | 130.83±13.66 | 129.56±19.06 | 0.769 | 147.74±44.61 | 151.12±47.30 | 0.799 |
| PLT (10^9^/L) | 290.88±89.39 | 290.31±96.09 | 0.971 | 277.13±100.78 | 318.55±103.19 | 0.109 | 294.66±151.54 | 238.87±97.28 | 0.128 |
| NEUT (10^9^/L) | 4.93±3.71 | 5.22±2.74 | 0.585 | 5.40±5.44 | 4.20±2.24 | 0.207 | 4.29±1.54 | 3.88±1.82 | 0.405 |
| LYMPH (10^9^/L) | 13.97±83.74 | 2.51±1.67 | 0.162 | 2.51±1.88 | 2.77±1.73 | 0.57 | 2.76±1.42 | 2.52±1.48 | 0.567 |
| MONO (10^9^/L) | 0.56±0.21 | 0.78±0.97 | 0.104 | 0.72±0.54 | 0.62±0.27 | 0.329 | 2.58±9.36 | 3.86±11.23 | 0.671 |
| NLR | 3.13±4.23 | 3.01±3.09 | 0.836 | 3.62±5.52 | 2.11±1.51 | 0.098 | 2.35±2.54 | 2.32±2.19 | 0.959 |
| MLR | 0.29±0.20 | 0.36±0.27 | 0.082 | 0.43±0.58 | 0.28±0.18 | 0.11 | 4.39±19.71 | 4.87±16.90 | 0.927 |
| PLR | 144.75±81.61 | 148.22±85.68 | 0.807 | 158.25±121.60 | 148.35±72.04 | 0.674 | 119.09±68.08 | 108.70±61.39 | 0.577 |
| Symptom | | | | | | |  |  |  |
| With | 29 (54.7%) | 39 (37.1%) | 0.035* | 15 (57.7%) | 11 (26.2%) | 0.009* | 13 (56.5%) | 11 (42.3%) | 0.331 |
| Without | 24 (45.3%) | 66 (62.9%) |  | 11 (42.3%) | 31 (73.8%) |  | 10 (43.5%) | 15 (57.7%) |  |
| Basic illness | | | | | | |  |  |  |
| With | 5 (9.4%) | 9 (8.6%) | 0.858 | 4 (15.4%) | 2 (4.8%) | 0.137 | 4 (17.4%) | 1 (3.9%) | 0.123 |
| Without | 48 (90.6%) | 96 (91.4%) |  | 22 (84.6%) | 40 (95.2%) |  | 19 (82.6%) | 25 (96.1%) |  |
| AFP (ng/mL) | | | | | | |  |  |  |
| <10 | 48 (90.6%) | 62 (59.1%) | <0.001* | 24 (92.3%) | 24 (57.1%) | 0.002* | 23 (100.0%) | 16 (61.5%) | <0.001* |
| ≥10 | 5 (9.4%) | 43 (40.9%) |  | 2 (7.7%) | 18 (42.9%) |  | 0 (0%) | 10 (38.5%) |  |
| β-HCG (mIU/mL) | | | | | | |  |  |  |
| <5 | 53 (100%) | 80 (76.2%) | <0.001* | 26 (100%) | 26 (61.9%) | <0.001* | 23 (100.0%) | 19 (73.1%) | <0.001* |
| ≥5 | 0 (0%) | 25 (23.8%) |  | 0 (0%) | 16 (38.1%) |  | 0 (0%) | 7 (26.9%) |  |
| Adler classification | | | | | | |  |  |  |
| 0-1 | 30 (56.6%) | 27 (25.7%) | <0.001* | 11 (42.3%) | 17 (40.5%) | 0.884 | 12 (52.2) | 7 (26.9%) | 0.073 |
| 2-3 | 23 (43.4%) | 78 (74.3%) |  | 15 (57.7%) | 25 (59.5%) |  | 11 (47.8%) | 19 (73.1%) |  |
| *, *P* < 0.05; BMI, body mass index; WBC, white blood cell count; RBC, red blood cell count; HGB, hemoglobin; PLT, platelet count; NEUT, neutrophil count; LYMPH, lymphocyte count; MONO, monocyte count; NLR, neutrophil-to-lymphocyte ratio; MLR, monocyte-to-lymphocyte ratio; PLR, platelet-to-lymphocyte ratio. | | | | | | | | | |

**Supplementary Table 5** The results of radiomics features selection.

| Key radiomics features for predicting testicular neoplasm | Key radiomics features for predicting testicular carcinoma |
| --- | --- |
| Feature name | Feature name |
| wavelet_HHL_firstorder_Mean | original_shape_Elongation |
| wavelet_LLL_glcm_ldmn | original_glcm_ClusterProminence |
| wavelet_HLL_glcm_ldmn | gradient_glszm_ZoneEntropy |
| wavelet_HHL_glcm_DifferenceVariance | wavelet_HLH_glrlm_LongRunEmphasis |
| wavelet_LHL_glszm_LargeAreaHighGrayLevelEmphasis |  |
| exponential _glszm_LargeAreaHighGrayLevelEmphasis |  |
| wavelet_LLH_gldm_LargeDependenceLowGrayLevelEmphasis |  |

**Supplementary Table 6** Univariate and multivariate logistic regression analysis of clinical features for discriminating testicular neoplasm from non-neoplasm in the training cohort.

| Variable | Univeriate analysis | | Multivariate analysis | |
| --- | --- | --- | --- | --- |
|  | OR (95%*CI*) | *P*-value | OR (95%*CI*) | *P*-value |
| Age (years) | 0.993 (0.991, 0.995) | <0.001* | 0.997 (0.995, 1.000) | 0.092 |
| BMI(kg/m^2^) | 0.996 (0.986, 1.005) | 0.441 |  |  |
| WBC (10^9^/L) | 0.995 (0.982, 1.007) | 0.485 |  |  |
| RBC (10^9^/L) | 1.064 (1.003, 1.129) | 0.082 |  |  |
| HGB (g/L) | 1.000 (0.998, 1.002) | 0.871 |  |  |
| PLT (10^9^/L) | 1.001 (1.000, 1.001) | 0.048* | 1.000 (1.000, 1.001) | 0.866 |
| NEUT (10^9^/L) | 0.976 (0.964, 0.990) | 0.004* | 0.981 (0.956, 1.007) | 0.222 |
| LYMPH (10^9^/L) | 1.001 (0.999, 1.002) | 0.498 |  |  |
| MONO (10^9^/L) | 1.024 (0.961, 0.985) | 0.542 |  |  |
| NLR | 0.973 (0.961, 0.985) | <0.001* | 0.999 (0.967, 1.033) | 0.958 |
| MLR | 0.789 (0.679, 0.917) | 0.010* | 1.081 (0.884, 1.322) | 0.520 |
| PLR | 0.999 (0.999, 1.000) | 0.006* | 1.000 (0.999, 1.001) | 0.999 |
| Symptom (with) | 0.752 (0.690, 0.820) | <0.001* | 0.811 (0.744, 0.884) | <0.001* |
| Basic illness | 0.774 (0.662, 0.907) | 0.008* | 0.898 (0.766, 1.052) | 0.263 |
| AFP (≥10 ng/mL) | 1.364 (1.242, 1.496) | <0.001* | 1.170 (1.057, 1.297) | 0.012* |
| β-HCG (≥5mIU/mL) | 1.303 (1.163, 1.461) | <0.001* | 1.229 (1.097, 1.377) | 0.003* |
| Adler classification | 1.067 (0.969, 1.175) | 0.262 |  |  |

OR, odds ratio; *CI*, confidence interval; *, *P*<0.05; BMI, body mass index; WBC, white blood cell count; RBC, red blood cell count; HGB, hemoglobin; PLT, platelet count; NEUT, neutrophil count; LYMPH, lymphocyte count; MONO, monocyte count; NLR, neutrophil-to-lymphocyte ratio; MLR, monocyte-to-lymphocyte ratio; PLR, platelet-to-lymphocyte ratio.

**Supplementary Table 7** Univariate and multivariate logistic regression analysis of clinical features for discriminating testicular malignant tumor from benign lesions in the training cohort.

| Variable | Univeriate analysis | | Multivariate analysis | |
| --- | --- | --- | --- | --- |
|  | OR (95%*CI*) | *P*-value | OR (95%*CI*) | *P*-value |
| Age (years) | 0.999 (0.996, 1.001) | 0.337 |  |  |
| BMI(kg/m^2^) | 1.006 (0.995, 1.016) | 0.393 |  |  |
| WBC (10^9^/L) | 1.000 (0.986, 1.014) | 0.999 |  |  |
| RBC (10^9^/L) | 1.035 (0.967, 1.107) | 0.406 |  |  |
| HGB (g/L) | 1.001 (0.998, 1.003) | 0.685 |  |  |
| PLT (10^9^/L) | 1.000 (1.000, 1.001) | 0.374 |  |  |
| NEUT (10^9^/L) | 0.997 (0.981,1.013) | 0.730 |  |  |
| LYMPH (10^9^/L) | 0.999 (0.998, 1.000) | 0.179 |  |  |
| MONO (10^9^/L) | 1.058 (0.982, 1.139) | 0.211 |  |  |
| NLR | 0.999 (0.975, 1.005) | 0.276 |  |  |
| MLR | 1.004 (0.842,1.197) | 0.969 |  |  |
| PLR | 1.000 (0.999, 1.001) | 0.939 |  |  |
| Symptom (with) | 0.816 (0.736, 0.906) | 0.002* | 0.838 (0.762, 0.922) | 0.003* |
| Basic illness | 0.896 (0.745, 1.078) | 0.326 |  |  |
| AFP (≥10 ng/mL) | 1.425 (1.278, 1.586) | <0.001* | 1.246 (1.115, 1.391) | 0.001* |
| β-HCG (≥5mIU/mL) | 1.533 (1.349, 1.742) | <0.001* | 1.366 (1.200, 1.556) | <0.001* |
| Adler classification (2-3) | 1.237 (1.113, 1.376) | 0.001* | 1.199 (1.089, 1.320) | 0.002* |

OR, odds ratio; *CI*, confidence interval; *, *P*<0.05; BMI, body mass index; WBC, white blood cell count; RBC, red blood cell count; HGB, hemoglobin; PLT, platelet count; NEUT, neutrophil count; LYMPH, lymphocyte count; MONO, monocyte count; NLR, neutrophil-to-lymphocyte ratio; MLR, monocyte-to-lymphocyte ratio; PLR, platelet-to-lymphocyte ratio.

**Supplementary Figure 1** Details of radiomics features extracted from ultrasound images.

**
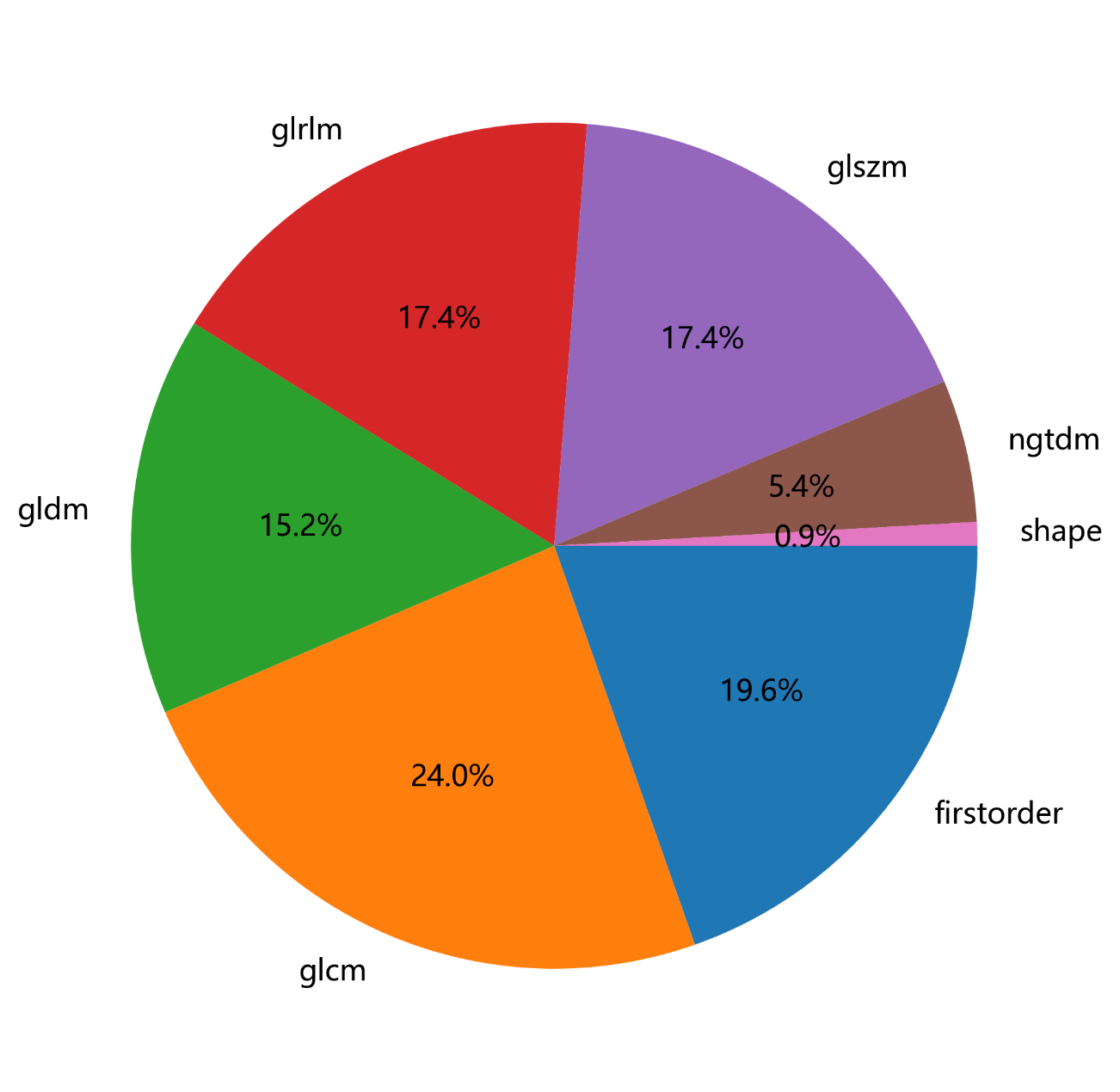
**
